# Analog Spatial Light Modulators Based on Micromirror Arrays

**DOI:** 10.3390/mi12050483

**Published:** 2021-04-23

**Authors:** Ulrike Dauderstädt, Peter Dürr, Andreas Gehner, Michael Wagner, Harald Schenk

**Affiliations:** 1 Fraunhofer IPMS, Maria-Reiche-Straße 2, 01109 Dresden, Germany; peter.duerr@ipms.fraunhofer.de (P.D.); andreas.gehner@ipms.fraunhofer.de (A.G.); michael.wagner@ipms.fraunhofer.de (M.W.); harald.schenk@ipms.fraunhofer.de (H.S.); 2 Chair of Micro and Nano Systems, Brandenburgische Technische Universität Cottbus-Senftenberg, Konrad-Zuse-Straße 1, 03046 Cottbus, Germany

**Keywords:** micromirror arrays, microlithography, microscopy, image generation, wavefront shaping

## Abstract

The Fraunhofer Institute for Photonic Microsystems (IPMS) has been developing and manufacturing micromirror arrays for more than 20 years. While originally focusing on applications related to microlithography and therefore mainly for light in the deep ultraviolet range, the range of applications has been expanded since, including applications in the visible and near-infrared range. This paper gives an overview of the devices and their designs, fabrication, and characterization.

## 1. Introduction

The pioneering work of Texas Instruments on “Digital Micromirror Devices” (DMD™) [1] and the improved quality and availability of other spatial light modulator (SLM) technologies, in particular liquid crystals on silicon (LCoS) [2], both mainly focused on home theater and business projector applications, as well as displays (from small displays of hand-held devices to large monitors), made SLM technology accessible also for a large variety of other applications, for example in the fields of metrology [3] or computer-generated holography [4].

However, there is also an increasing number of other applications as diverse as image generation, wavefront shaping, deep tissue imaging, material processing, etc., that require wavelength ranges beyond that of commercially available SLMs or real-time grayscaling at high frame rates. In these cases, custom-made SLMs are required, which can meet some or all of these requirements.

SLMs based on micromirror arrays (MMAs) are now available in very different shapes and sizes. In 2018, Song et al. published a very comprehensive review of available devices [5]. Among the analog two-dimensional MMAs introduced, there are for example those by Boston Micromachines, who have been active in the field of membrane SLMs [6], as well as increasingly large micromirror arrays with piston-tip-tilt actuators [7]. Then, there is Silicon Light Machines, which has worked mainly on one-dimensional SLMs based on the grating light valve technology (GLV™) [8], but introduced also a two-dimensional array in 2003 [9]. Furthermore, there is the IMEC in Belgium with the largest MMA that has been published so far (11 million mirrors of 8×8µm) [10].

The work on light valve technologies on which the work presented here was based started in the late 1980s at the Heinrich-Hertz-Institut für Nachrichtentechnik in Berlin [11]. It was continued at the Fraunhofer Institute for Microelectronic Systems (IMS), of which the current Fraunhofer Institute for Photonic Microsystems (IPMS) was then a part. The early work at the IPMS was focused on microlithography. This means that much work has been done with respect to the use of micromirrors in ultraviolet (UV) and deep ultraviolet (DUV) light. Although within the early 1990s, the main SLM activities were dedicated to viscoelastic control layers with reflective coatings, the first work was done on free-standing MEMS micromirror arrays [12], and by the late 1990s, the transition was made to micromirrors; see [13,14].

Since then, more types of mirrors and arrays have been developed including devices for applications in the visible and near-infrared (NIR) spectral range.

Most of these devices have in common that they can operate in analog mode, i.e., the mirror deflections can be set to specific individual values, thus allowing for real-time grayscaling.

## 2. Types and Applications

There are several basic types of mirror currently being developed at the IPMS: one-axis tilting mirrors, which are rotated around one axis, two-axis tilting mirrors, which can be tilted around two axes and therefore in any direction, and piston mirrors, which are translated in a direction perpendicular to the mirror surface. In the following, these types and their applications are briefly described.

### 2.1. One-Axis Tilting Mirrors

**Figure 1:**
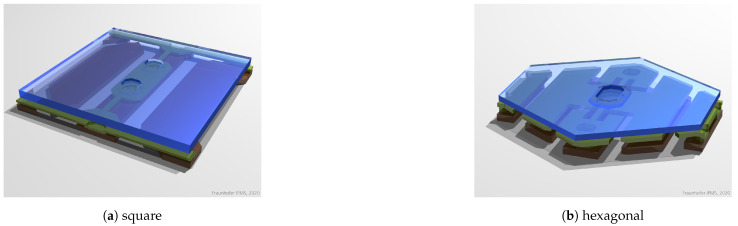
One-axis tilting mirrors.

Among the first micromirror arrays developed at the IPMS were tilting mirrors for image generation [15]. A drawing of them can be seen in Figure 1a. The mirrors are $16\times 16\;µm^{2}$ in size. The actuator consists of three functional layers:

1 The electrodes (bottom layer, brown):
  (a) The mirror electrode (center): provides a common voltage for all mirrors, used mainly as a bias (set to a negative voltage) to increase the electrostatic force between the mirror and the address electrode.
  (b) The counter electrode (right): used to compensate for positive pre-deflections, identical for all mirrors.
  (c) address electrode (left): provides the individual address voltages.
2 The “hinge” layer (second layer, green): This contains the torsional springs that provide the force to counterbalance the electrostatic force. The springs are connected electrically, as well as mechanically to the mirror electrode by posts. In addition, in the example shown in Figure 1a, there are bars perpendicular to the springs at the upper and lower edge that prevent the mirror from touching either the counter or address electrode and thus causing short cuts. In the example shown in Figure 1b, the same purpose is served by landing electrodes that run between the mirrors.
3 The mirror (top layer, blue).

**Figure 2:**
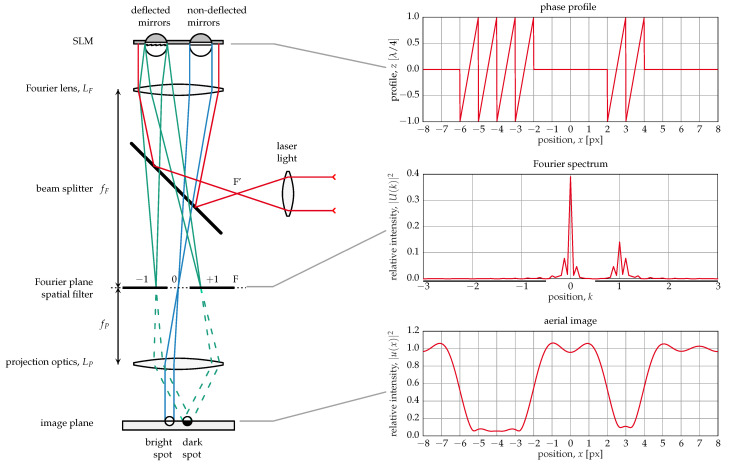
Fourier optical imaging principle.

These mirrors were developed for a lithography application in the DUV spectral range (248 nm) [16]. The imaging principle is illustrated in Figure 2. Light from a coherent light source—in this case, an excimer laser—passes through a beam splitter and a lens onto the mirror array (spatial light modulator (SLM)). The SLM acts as an optical grating, where light from flat, i.e., non-deflected mirrors, is reflected into the zeroth order while light from deflected mirrors goes (completely or partially, depending on the deflection) into higher orders. This process is mathematically described by a Fourier transform of the phase in the mirror plane. A spatial filter in the Fourier plane blocks all but the zeroth order, and another lens (projection optics) performs the back transformation, yielding an image with bright and dark spots corresponding to flat and deflected mirrors, respectively. The mirror deflection required for all light to be moved from the zeroth to the first diffraction order is a quarter of the wavelength, λ/4, for this kind of mirror. If the deflection is smaller, some light will still be in the zeroth order, resulting in a gray pixel. Thus, this principle can be used to create real grayscale images. However, the requirements with respect to the accuracy and stability of deflection, as well as mirror planarity are much higher than when much larger deflections are used, as with the digital mirror devices (DMD™) by Texas Instruments, where the mirrors have only two states and gray values are created by pulse width modulation (PWM) [1].

The device used for this application has 512×2048 mirrors with a size of $16\times 16\;µm^{2}$; see Figure 6. The image can be updated at a frame rate of 2 kHz.

Similar devices with a smaller number of mirrors (256×256) have been developed for use in the visible and near-infrared spectral range, for example for applications in microscopy [17,18].

A hexagonal version of the tilting mirrors as shown in Figure 1b was developed for a lithography application at 355 nm [19]. These mirrors have a yoke in the hinge layer so that the electrostatic force acts between the electrode and the yoke instead of the mirror, which gives more freedom in the choice of material for the mirror and the yoke, as well as their design. The different design of the yoke allows for a smaller gap between yoke and electrode, leading to a higher electrostatic force, which in turn allows for stiffer hinges, resulting in a higher resonant frequency. The hexagonal shape of the mirror results in a reduced sensitivity to mirror non-planarity.

### 2.2. Two-Axis Tilting Mirrors

There are applications where it is not desirable to block the light of the higher diffraction orders, but to use (almost) all light and only re-distribute it. These would be cases where relatively large laser powers are needed like laser material processing, e.g., for laser ablation, graving, or cutting, or laser spot steering, e.g., for optical tweezers. In such applications, one would not want to waste light and also keep the energy, to which the SLM is exposed, low.

**Figure 3:**
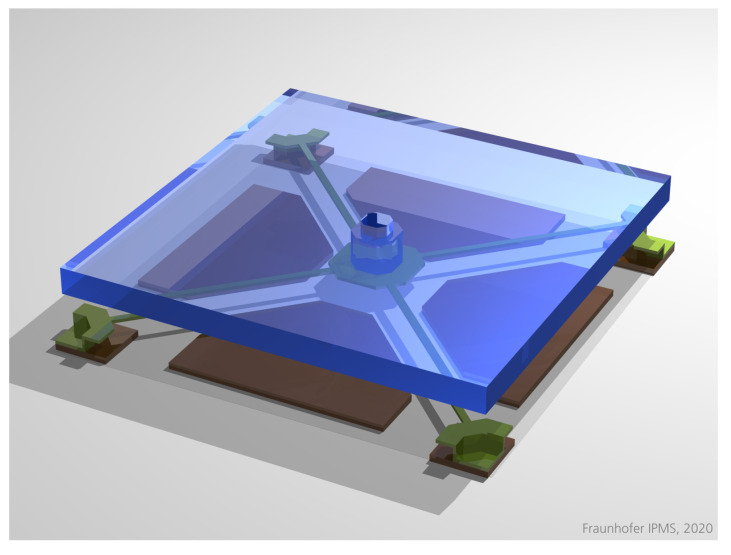
Schematic image of a two-axis tilting mirror.

A schematic image of a mirror used for this is shown in Figure 3, and more detail is provided in [20]. These mirrors cannot only be tilted around one axis, but in any direction. This is achieved by placing four address electrodes underneath the mirror.

In this case, the formation or modulation of intensity patterns is performed in the SLM’s Fourier plane and not within its conjugated image plane as in light valve projection. This is suitable for applications with images of relatively low resolution and high laser intensity. The principle has been demonstrated by using these mirrors for writing markings on a steel plate.

### 2.3. Piston Mirrors

A large range of applications for micromirror arrays exist as in optical wavefront control in adaptive optics or in holography. For these applications, piston mirrors are better suited than tilting mirrors. In the field of wavefront correction, more often, membrane mirrors [21,22] or piston-tip-tilt mirrors [23] are used. However, these usually have much fewer elements than the arrays discussed here (a few dozen to a few thousand instead of several ten thousand to millions).

In the case of wavefront control, the mirrors may be comparatively larger as for image formation, e.g., up to 100 µm and beyond, to facilitate sufficiently large phase amplitudes in terms of stroke. In the early stages, we obtained up to a 1.2 µm stroke with 100 µm pixels [24], which meanwhile could be extended up to 2.5 µm even with a smaller 40 µm pixel size.

On the other hand, mirror arrays for computer-generated holography, on which work has just begun in a project funded by the European Union (REALHOLO, Horizon 2020 Grant Agreement No. 101014977, https://realholo.eu/, accessed on 17 April 2021), have very different requirements: the mirrors need to be much smaller (a few micrometers) and the arrays much larger (several millions of mirrors).

### 2.4. Mirror Addressing Circuit (Backplane)

**Figure 4:**
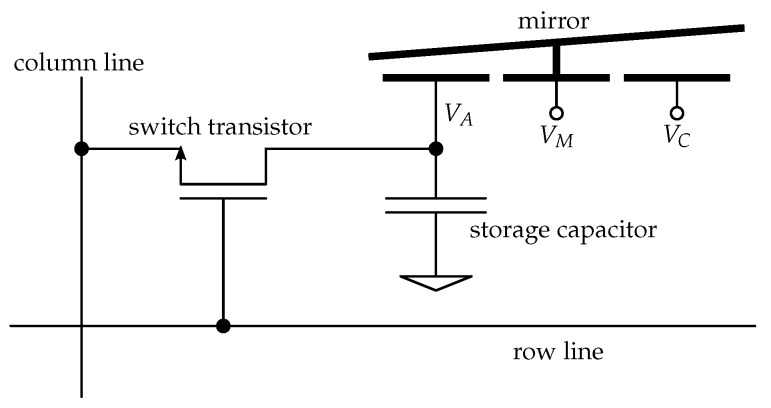
Schematic of a pixel cell.

The voltages required to address the mirrors are provided by DRAM-like storage cells connected to the address electrode(s) of the mirror [25]. Figure 4 shows an electronic schematic of a pixel with a one-axis tilting mirror. The voltages $V_{M}$ and $V_{C}$ are supplied directly and are identical for all mirrors.

For the piston mirrors, the address voltage is applied to a single electrode underneath each mirror while the mirrors are all connected to a common voltage.

For the two-axis tilting mirrors, four individual address voltages are provided for each element, while the mirrors are again set to the same common potential.

## 3. Improving Mechanical, Thermal, and Optical Properties Based on Simulations

In order to better understand the behavior of the SLMs and the effects involved in their operation, as well as to save time and costs for the development and fabrication of the devices, extensive simulations are being performed. This starts with both analytical and numerical (FEM) simulations of the mechanical behavior of the mirrors and hinges like mechanical sensitivity, resonant frequency, and damping behavior. Another aspect would be thermal behavior due to heat generation by the electronics or light absorption by the mirrors. These effects are important for mirror and chip planarity. Furthermore, optical simulations specific to the application are performed in order to understand the customers’ needs and be able to cooperate in the development of specifications and interfaces.

## 4. Fabrication

### 4.1. CMOS Fabrication

The basis for the fabrication of the IPMS SLMs is a high-voltage CMOS process developed at Fraunhofer IMS, early versions of which were described in [11,14,26]. This allows providing up to several million mirrors with address voltages up to ≈26V at a frame rate of up to several kHz. The devices discussed here have several ten thousand up to a million active mirrors that can be operated at frame rates up to about 5 kHz at present.

### 4.2. Mirror Fabrication

**Figure 5:**
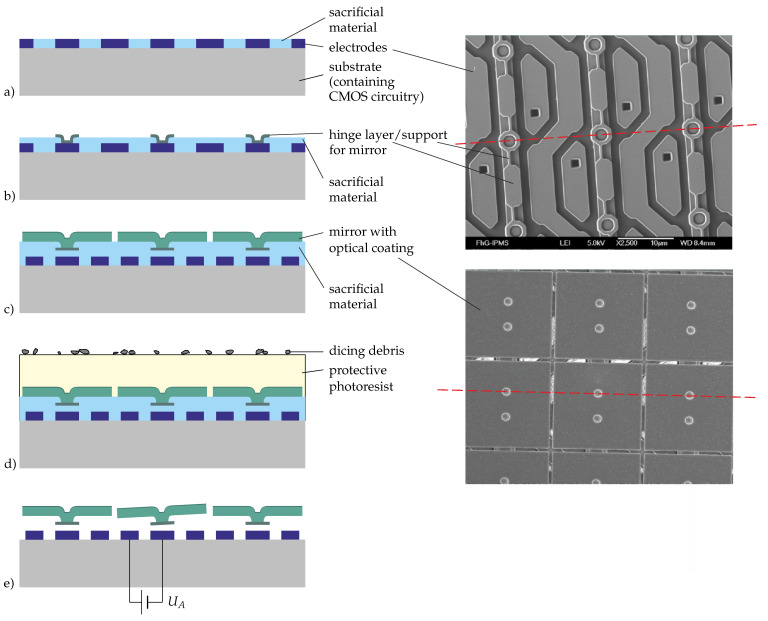
Fabrication of micromirror arrays: on the left, drawings of cross-sections indicating the main steps of the fabrication are shown; on the right, there are SEM images of the electrode and hinge layers (top) and the mirrors (bottom); the red lines indicate the position of the cross-sections in the drawings (the line in the upper SEM image referring to (**a**,**b**) and in the lower one to (**c**–**e**).

The fabrication of the actuators and mirrors is shown in Figure 5. Starting with the complete CMOS part and the address electrodes, an inorganic sacrificial material is deposited and planarized by means of chemical mechanical polishing (CMP) in order to obtain a smooth surface for the actuator (Figure 5a). On this, the first sacrificial layer and the hinge material are deposited and patterned (Figure 5b). After the deposition, polishing, and patterning of the second sacrificial layer, the mirrors are deposited and patterned (Figure 5c). To protect the mirrors during dicing, a photoresist is deposited (see Figure 5d), which is removed by a solvent after the dicing. Finally, all the sacrificial material (including that between the electrodes) is removed in a vapor etching step, leaving free-standing mirrors (Figure 5e).

The technology for the other mirror types discussed above is in principle the same. In some cases, a shield layer below the electrodes is used in order to protect the memory cell underneath from the light.

### 4.3. Die Bonding

Since the global surface planarity of the mirror area is an essential parameter, the die bonding process is of great importance. It had to be optimized in order to achieve both sufficient absolute planarity and stability. Both parameters depend on the planarity of the chip and the substrate onto which the chip is bonded, sometimes on the total thickness variation of the chip, as well as the properties of the glue used for die bonding.

Die bonding with a large amount of low viscosity glue leads to relatively good initial planarity (especially if a spherical shape is acceptable) even if the substrate is not particularly flat, like the cavity of a ceramics package. Here, the shape of the chip prior to die bonding is largely preserved even after hardening (and shrinking) of the glue. However, the glue thickness after hardening is not stable, especially if the humidity of the environment changes after the die bonding [27].

**Figure 6:**
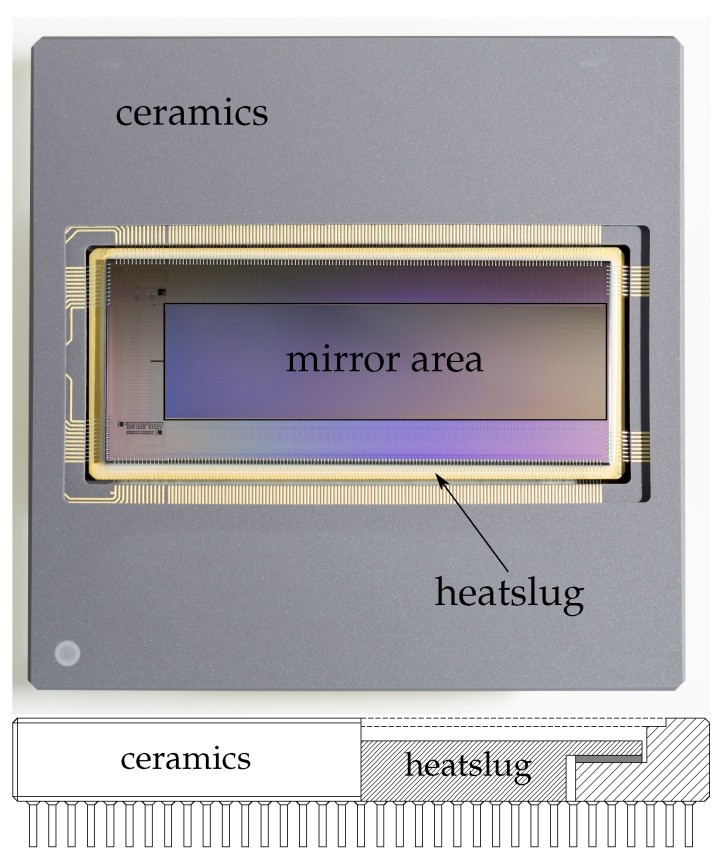
Ceramics package with heat slug and chip: On the top is a photograph of the package with the chip, and on the bottom is a drawing of a horizontal cross-section through the center of the package (without the chip).

In order to increase the stability, the amount of glue can be reduced. However, in this case, the planarity of the substrate becomes more important. A good, practical solution is the use of ceramic packages with a so-called “heat slug” in the middle, which can be made with a very good planarity; see Figure 6. By using this, a good compromise between initial planarity and stability (since very little glue is used) can be found, which is sufficient even for the more demanding applications so far. Additionally, the heat slug provides a good thermal connection between the chip and the environment. In this way, the planarity of a few hundred nanometers (peak-to-valley) or less can be achieved on mirror areas of several centimeters in length; see Figure 11.

If the global planarity and/or its stability are to be further improved, the thickness variation of the chip has to be taken into account. If, for example, eutectic bonding to a very flat surface is used, one would get a near-perfect stability of the global flatness, but the absolute values might actually get worse than for glued chips. For this approach to work, a map of the chip’s thickness variation would be required, the inverse of which would have to be transferred onto the substrate and the chip would have to be positioned with sufficient accuracy during bonding. A somewhat similar approach was suggested in [28], where the effect of the glue shrinking during hardening was used. It has been shown that this does work in principle. Furthermore, an approach using piezoresistive actuators to pull the active area flat could be considered. However, all three approaches come with considerable cost both in R&D and fabrication so that they will only be implemented if necessary.

### 4.4. Sealing the SLM

The die bonding process as described in the previous section leaves the mirror surface open to the environment. Therefore, measures have to be taken to protect it from:

- direct mechanical damage
- particles
- humidity
- corrosive substances
- oxygen

The last item is particularly important when the mirrors are operated in DUV light, where the ozone created by the light in combination with the electrical fields underneath the mirrors oxidized the surface of the address electrodes, leading to trapped charges in the oxide and thus a reduced electrostatic force acting on the mirrors; see [29].

**Figure 7:**
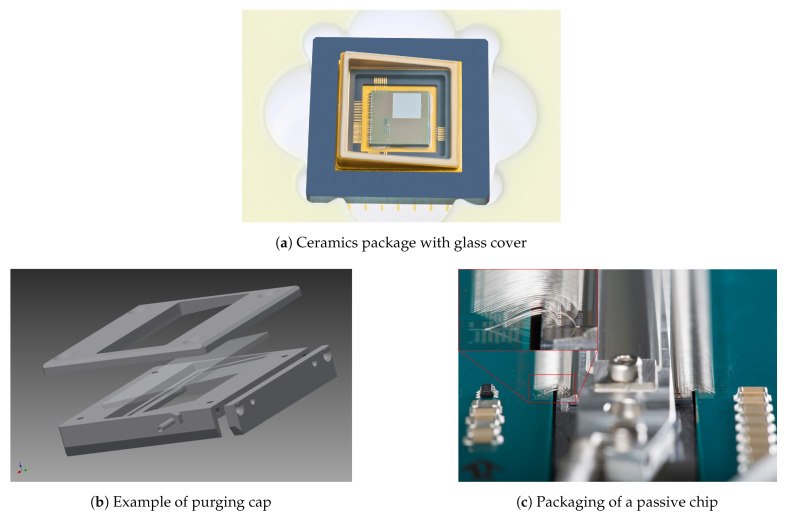
Examples for packaging the SLM.

Since the encapsulation has a significant impact on the optical performance of the SLM, it has to be adjusted to the application for which the SLM is to be used. The encapsulation can be done by gluing or soldering glass on top of the ceramic package (Figure 7a) or mounting a purging cap to the package (Figure 7b). The latter allows the purging of the SLM with an inert gas, usually dry nitrogen. Particle filters are attached to keep the gas clean. At present, the possibilities for a hermetic sealing of the encapsulation are being explored as well.

Usually, the glass is tilted in order to remove light reflected by the glass from the optical path.

A very special case is shown in Figure 7c. This is a passive device, i.e., one without the CMOS circuit underneath, where 8193 logical pixels have to be connected [19]. The SLM chip (top center, mirror area underneath a purging cap) is connected to a PCB with four loops of bond wires on top of each other. This process was developed in cooperation with a local company, which specializes in microchip assembly processes.

## 5. Characterization

In the following, a number of parameters that are essential for the function of the mirrors (in their specific application) and their characterization are described.

### 5.1. Local Planarity

Local planarity means the planarity of individual mirrors and consists of a number of parameters (pre-deflection, bow, higher order Zernike modes, roughness). Depending on the specific applications, especially the wavelengths in which the mirrors are operated, the requirements with respect to mirror planarity can be very high, with RMS values down to a few single nanometers. Therefore, many resources are spent in order to investigate mirror planarity, after fabrication, over time, and during operation.

Local planarity is mostly measured by means of optical profilometry. Modern, high-end profilometers allow automated profile measurements with a vertical resolution of ≈0.1 nm and a lateral resolution of ≈0.33 µm (which is near the optical limit). The tools allow both phase shift interferometry (PSI) and vertical scanning interferometry (VSI) or white-light interferometry. The PSI mode allows the best resolutions (both vertical and lateral) and accuracy and is used for actual planarity measurements, while the VSI mode is less accurate, but works for discontinuous surfaces, i.e., deflected mirrors, and is therefore used for deflection measurements (including calibrations).

A stroboscopic illumination is available for some tools to allow quasi-dynamic measurements.

The interferometers are placed in a cleanroom lab with a separate foundation so that there is minimal disturbance due to vibrations.

**Figure 8:**
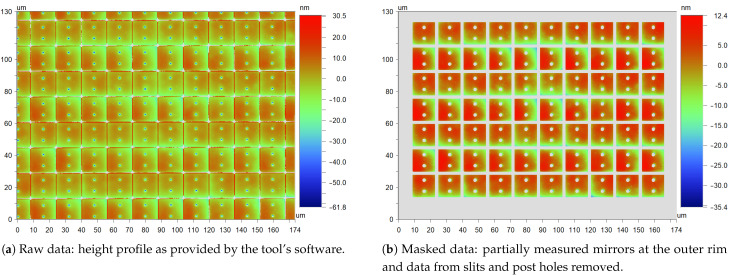
Local flatness measurement by means of an interferometer.

Figure 8a shows an example of a profile measurement of the 16 µm mirrors. This image includes the relevant data for the mirrors, but also data from the slits and post holes. Those are often very inaccurate since very steep edges cannot be measured well by means of an interferometer. Therefore, they have to be removed from the data set.

Thus, in the next step, individual mirrors are identified in the image by means of pattern recognition (using proprietary IPMS software), resulting in the image in Figure 8b, where all mirrors are clearly separated and the data from slits and post holes are removed.

After that, a plane is fitted to each individual mirror, yielding values for mirror deflection in the *x*- and *y*-direction, as well as the vertical position. These data are then used to remove the overall tilt of the image, which is slightly different from the tilt calculated by the tool’s software since partial mirrors at the edges are removed. Finally, several parameters describing mirror planarity are calculated for each mirror by fitting the appropriate functions to the data. These data are then summarized as means and standard deviations for a measurement field or used separately.

To give an example of the results, for mirrors used in DUV imaging systems, the RMS value, i.e., the standard deviation from the plane, is typically in the range of 2 nm to 4 nm at a mirror size of $16\times 16\;{µm}^{2}$. Most of the variation is due to process variations and differences between wafers and batches, and the variation on a chip is usually in the subnanometer range.

### 5.2. Mirror Calibration for a Precise Analog Deflection Control

In order to be able to use grayscaling, the deflection of the mirrors needs to be controlled quite accurately, in some cases in the range of 0 to 0.25λ (deflection being the height difference between the center and edge of the mirror); in other cases, it is several degrees in one or more directions. Due to mechanical stress in the hinges and between the hinge and mirror, as well as the non-planarity of the sacrificial layer, all mirrors have an initial deflection other than zero. Furthermore, their response to a voltage varies due to variations in hinge width and thickness, the distance between the mirror and electrodes (caused by variation of sacrificial layer thickness and initial deflection), etc. Therefore, it is necessary to adjust the address voltage for each individual mirror.

**Figure 9:**
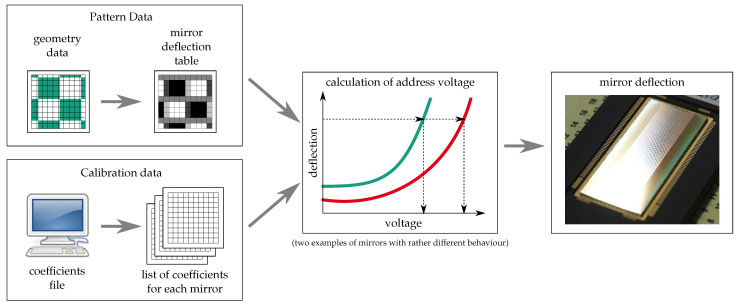
Schematic representation of the calibration procedure as described in [30].

This is done by measuring of the deflection characteristic of each mirror again using optical profilometry. With these data, a number of global and individual fit parameters are calculated. Those are stored in a calibration file for each chip. This part of the process is illustrated by the box in the lower left part of Figure 9.

In most image-generation applications we have dealt with so far (industrial applications in DUV microlithography), grayscaling is used to fine-position the edges of patterns. In order to achieve this, a gray value is calculated for each pixel that corresponds to the relative area of this pixel covered by the feature that is to be written. This is illustrated in the box on the upper left part of Figure 9.

The information from these two parts is then combined to choose the correct voltage for each pixel (Figure 9, center and left).

**Figure 10:**
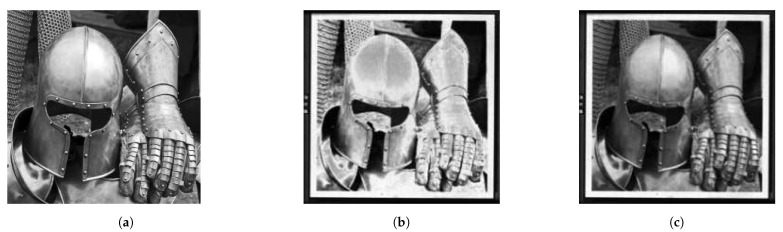
Calibration result based on optical profilometry [30]. (**a**) Original image. (**b**) Camera image taken with uncalibrated mirrors, the wrong gray values, and noise due to the spread in mirror response, as well as systematic predeflection. (**c**) Camera image taken with calibrated mirrors.

Figure 10 illustrates the effect of the calibration. Figure 10a shows the image that was used as the input data. Figure 10b shows a camera image for an uncalibrated chip. Due to the spread in the mirror response, as well as a systematic pre-deflection, the latter causes, for example, the center of the top of the helmet to be darker instead of brighter than the surrounding areas. Figure 10c finally shows the camera image for a calibrated chip. The gray values now correspond quite well to those in the original image, and the features are much better resolved.

Although this method can control the deflection quite accurately, it does not take care of effects caused by mirror planarity and slits and thus results not necessarily in the best possible contrast. However, it is sufficient for many applications. If a better accuracy is required, the optical response of the mirrors needs to be used for calibration; see Section 5.4. In this way, it is possible to partially compensate for the influence of light from the slits by adjusting the deflection.

### 5.3. In Situ Planarity Measurements

In our early attempts to analyze the behavior of our mirrors when used in DUV light, it became clear that it was not sufficient to expose the mirrors to the light and analyze them before and after, but that they had to be observed during mirror operation in DUV light. In order to achieve that, an optical profilometer was built, which allows profilometric measurements during DUV exposure [31]. Naturally, this tool has not quite the same resolution, accuracy, and degree of automation as the commercial tools, but is quite flexible and well suited to analyze mirror behavior under real operation conditions.

### 5.4. Contrast Measurements

In order to be able to investigate the behavior of the SLMs in an optical system, a setup was built to demonstrate the optical principle discussed above. However, this setup uses mirrors instead of lenses so that light sources of significantly different wavelengths can be used; see [30]. With this setup, it has been possible not only to measure characteristics like contrast [32], but also to calibrate the mirrors using their optical behavior. This has the advantage that not only effects caused by inaccurate deflection are corrected, but also errors caused by planarity or slits can be compensated to some extent.

Thus, while the calibration of deflection using interferometry allows contrast values of up to 1000–2000, which can be more than ten times as much as for the uncalibrated case [30], using this optical calibration can increase contrasts to more than 10,000 [33].

However, the contrast is by no means the only relevant optical parameter. In microlithography, for example, the accuracy of the achieved gray level and the spatial resolution are just as important, which are, just as the contrast, properties not only of the micromirror array, but of the whole optical setup, although the mirrors are a limiting factor. The optical calibration has been shown to yield a mean difference from the target value (50% gray) of 0.06% with a standard deviation of 0.07% [34].

### 5.5. Global Planarity

As discussed in Section 4.3, global planarity is an important parameter since variations in the surface profile of the chip can lead to focus variations and distortions of the image. It is also measured by interferometry, specifically a Fizeau interferometer with a large objective. This allows the complete mirror area to be imaged in one measurement. In these measurements, the influence of vibrations is particularly critical; therefore, in addition to the tool being placed in a low-vibration environment (see above), it is configured so that the samples are placed upside-down on top of the objective so that the two cannot move with respect to each other during measurement.

**Figure 11:**
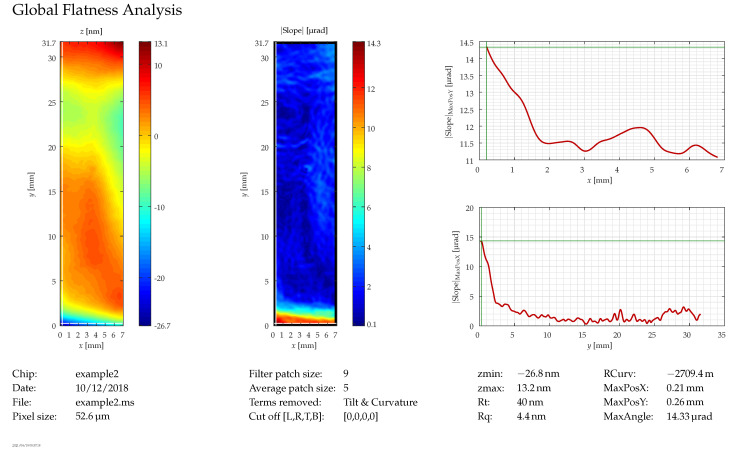
Global planarity: the white lines in the left image (at the left and bottom, intersecting at the point with the highest slope) indicate the position of the curves on the right.

After the measurement is taken, the data are post-processed to smoothen the profile by means of a Savitzky–Golay filter, extract the tilt and curvature and calculate slope, as well as the remaining non-planarity. Depending on the application, sometimes the relevant parameter for the global planarity is just the peak-to-valley value, which is in the order of a few hundred nanometers on a chip of 32 mm in length. However, since the remaining non-planarity usually has a significant spherical component, which can to some extent be compensated by optical means, the curvature is often subtracted and the angular deviation from the sphere calculated. This usually yields values of several ten microradians. Figure 11 shows a rather good example of these measurements with the height profile (after removal of the sphere) on the left, the angular deviation from the sphere (slope) in the center, and curves for the slope on cross-sections of the chip along the thin white lines at the left ( 0.21 mm ) and the bottom ( 0.26 mm ) of the chip, which run through the position of the largest value for the slope (MaxAngle).

Even though great care has been taken to eliminate vibrations from the measurement environment, there are still some left, which cause the “ripples” that can be seen in the upper left half of the image for the slope and in the lower curve (|Slope(y)|) between 15 mm and 25 mm. However, these disturbances are much smaller than the actual feature that can be seen at the bottom of the chip, where there is a real deformation. Thus, the smallest non-planarity that can be distinguished from the effect of the vibrations is about 5 µrad.

### 5.6. Measurement of Dynamic Properties

In order to be able to operate the mirrors at frame rates of several kilohertz and more, it is important to analyze their dynamic behavior, i.e., resonant frequency and damping behavior. In some cases, especially with larger mirrors, mirror deformation due to their movement also has to be analyzed.

**Figure 12:**
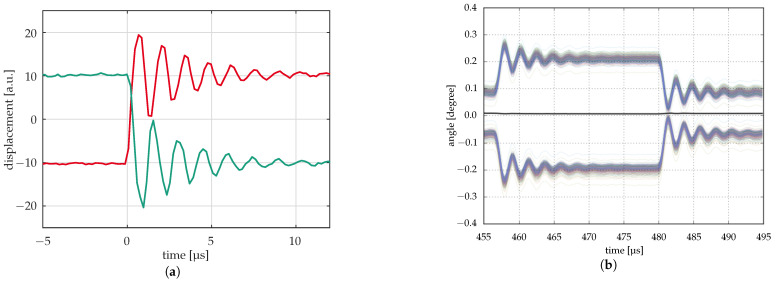
Dynamic measurements. (**a**) Early measurements of mirror response by laser vibrometer (two mirrors); (**b**) Measurement of mirror response by optical profilometry with stroboscopic illumination (ca. 450 mirrors) [35].

Real dynamic measurements are done by laser vibrometer (Figure 12a). Here, the movement of one or several points on a mirror is analyzed, providing data on position, velocity, and acceleration. This can be done with a high temporal resolution (several ten megahertz). However, the lateral resolution is limited, and there are some issues with drift in the measurement system.

If, however, a periodic behavior is to be analyzed, like the transient of the mirrors in pulsed operation, the optical profilometers can be used with a stroboscopic illumination, allowing measurements with high vertical, lateral, and temporal resolution with high accuracy and for many mirrors at once. Furthermore, all analysis procedures that have been developed for the optical profilometers at the IPMS can be applied here. Some of the commercial tools available have the option of a stroboscopic measurement, but not all. For the latter, a stroboscopic illumination module has been developed at the IPMS. This is a standalone illumination module that can be operated with different interferometers and different objectives, as long as the objective supports SMA fiber coupling. This allows the dynamic measurement of periodic events with a temporal resolution of up to several hundred kilohertz; see [35].

Figure 12b shows a comparison with similar measurements on the transient of 16 µm micromirrors using a laservibrometer (a) and an optical profilometer with stroboscopic illumination. The former shows the transients of two micromirrors with different initial deflection (t<0) and the opposite direction of deflection, measured separately. The optical profilometer shows about 450 curves for as many mirrors, half of which are deflected in the opposite direction of the others. This measurement takes much longer than the measurement with the laser vibrometer since each time step corresponds to a single measurement of a few seconds. Therefore, it works only if the transients always look the same and do not change significantly during the measurements. However, if this requirement is met, a large number of mirrors can be measured in parallel with high accuracy and with correct deflection differences with respect to each other. Furthermore, the shape of the mirrors during the transient can be measured with a high spatial resolution comparable to that in Figure 8b.

## 6. Summary

In this paper, the design, fabrication, and characterization of mirror arrays developed at the Fraunhofer IPMS were described, which allowed us to provide SLMs that met the high requirements of a number of diverse applications.
